# Relationship of resting heart rate and physical activity with insulin sensitivity in a population-based survey

**DOI:** 10.1186/s40200-015-0161-2

**Published:** 2015-05-06

**Authors:** Andrew Grandinetti, David MKI Liu, Joseph Keawe‘aimoku Kaholokula

**Affiliations:** Department of Public Health Sciences, John A. Burns School of Medicine, University of Hawai‘i at Manoa, 1960 East–west Road D-104 L, Honolulu, HI 96822 USA; Department of Native Hawaiian Health, John A. Burns School of Medicine, University of Hawai‘i at Mānoa, Honolulu, HI 96822 USA

**Keywords:** Asian Americans, Oceanic ancestry group, Insulin resistance, Cardiovascular, Epidemiology

## Abstract

**Background:**

Resting heart rate (RHR) has been identified as an independent risk factor for cardiovascular disease and mortality, contributing to atherosclerosis, the progression of heart failure, and myocardial ischemia and infarction. This study examines the association RHR and physical activity has with insulin resistance and insulin secretion in a multiethnic cohort from North Kohala, Hawai‘i.

**Methods:**

Cross-sectional data from 1,440 participants of Native Hawaiian, Japanese, Filipino, Caucasian, and mixed ethnic ancestries were analyzed for the study to include anthropometric measurements, and biochemical markers. Body fat was estimated by calculating body mass indices (BMI); body fat distribution by waist-hip ratios (WHR); and fasting plasma glucose and insulin levels were used to calculate insulin resistance using the Homeostasis Model (HOMA-IR). First phase insulin response was estimated using the insulin secretion ratio (ISR). Associations were estimated using general linear models (GLM).

**Results:**

Caucasians had lower mean RHR than all other ethnic groups; there were no statistically significant differences between other ethnic groups on mean RHR. HOMA-IR was associated with ethnic group, BMI and WHR, PA and RHR, while ISR was associated with age, ethnic group and BMI, but none of the primary risk factors. Both RHR and physical activity level remained significant for insulin resistance.

**Conclusions:**

In a multiethnic cohort from a rural community in Hawai‘i, increased RHR and a lower level of physical activity were both independently associated with increased risk for the development of insulin resistance, suggesting cardiovascular fitness may be as important as physical activity in preventing insulin resistance.

## Background

Previous studies have identified resting heart rate (RHR) as an independent risk factor for cardiovascular disease and mortality [1-6]. An increased resting heart rate may eventually result in the development of atherosclerosis, the progression of heart failure, and myocardial ischemia and infarction [7-9]. The mechanisms involved range from increased shear stress, cardiac noradrenaline synthesis, reduced arterial elasticity, decreased diastolic duration and coronary perfusion time, and plaque rupture. One study identified a 40% increased risk of all-cause mortality, and more than double the risk of heart failure hospitalization, for a resting heart rate ≥ 70 [10]. In patients with stable coronary disease and type 2 diabetes (DM 2), a 10 beats per minute increase in RHR was associated with decreased survival in patients with DM 2.

Several studies have demonstrated an association between elevated RHR and insulin resistance [11-14]. Insulin resistance is associated with compensatory hyperinsulinemia, and insulin has direct functions in the heart other than regulating glucose uptake [15]. Insulin itself is associated with increased sympathetic drive [16,17], resulting in elevated RHR and decreased heart rate variability. While some studies implicated hyperinsulinemia as the cause of increased RHR, two studies have correlated elevated baseline heart rate with the subsequent development of obesity and DM 2 [18,19].

Few studies have examined the independent roles of RHR and PA with insulin resistance. The Insulin Resistance Atherosclerosis Study (IRAS) [20] reported that insulin resistance and insulin secretion were associated with RHR, after adjusting for PA using the Modifiable Activity Questionnaire [21]; however, they did not report the effect of PA nor did they examine the potential interaction of PA and HR. The objective of the study reported here is to examine the independent relationships of both RHR and physical activity, and their interaction, with insulin resistance and beta-cell function among non-diabetic participants of the Kohala Health Study in North Kohala, Hawai‘i. To our knowledge, this is the first study examining this relationship in a multiethnic cohort composed of individuals of primarily Asian and Pacific Islander ancestry.

## Methods

The participants in this study were drawn from the Kohala Health Study (KHS). The KHS was a community-based, epidemiological study of cardiovascular and metabolic risk factors in the North Kohala district of the island of Hawai‘i. Over 1,500 residents of this multiethnic community participated, of which 1452 completed the entire examination and survey. Informed consent was obtained from each individual before participating in the study, which was approved by the University of Hawaii at Manoa’s Committee on Human Studies.

The methods of the KHS are described elsewhere [22]. In brief, participants fasted (with the exception of water) for 10–14 hours prior to the appointment. The clinical examination took approximately 2–3 hours after signed informed consents were obtained. Participants who were currently under treatment for diabetes did not undergo a 2-hour oral glucose tolerance test and were excluded from this analysis (n = 1327).

Blood was drawn in the fasting state and after a 75-gram oral glucose challenge. Plasma drawn from EDTA tubes were separated within 2 hours and one aliquot was stored at 4°C for lipid analyses within 7 days and the other aliquot frozen at −80°C for hormone analyses. The plasma concentrations of insulin were determined by radioimmunoassay (RIA) in duplicate. Insulin assays were performed using kits from Linco Research Inc. (St. Charles, MO). All measurements were performed with quality control procedures in place. Our laboratory also participated in the Center for Disease Control (CDC)-NHLBI lipid standardization program. Intra-assay and inter-assay coefficient of variances were all less than 10%.

Insulin resistance was estimated using the homeostasis model assessment for insulin resistance (HOMA-IR), which has a high correlation with the euglycemic-hyperinsulinemic clamp method, but is less costly and technically demanding to use in large scale epidemiological studies [23]. The HOMA-IR for molar units of glucose is calculated thusly: glucose × insulin/22.5. First phase insulin response was estimated using the insulin secretion ratio (ISR), which has been shown to be highly correlated with direct measurements of stimulated insulin secretion. The insulin secretion ratio is calculated as the ratio (30–0 minute fasting insulin)/(30–0 minute plasma glucose).

A medical history was obtained from each subject including history of diabetes, history of hypertension and taking medication for hypertension. Systolic and diastolic blood pressures (SBP, DBP) were recorded as the average of the second and third measurements. Anthropometric measurements were obtained while standing. Waist circumferences were measured at the level of the navel and used as an estimate of central adiposity. Height and weight were measured with participants wearing light-weight clothing without shoes, and used to calculate body mass indices [BMI; (kg/(m^2^)].

Past week leisure physical energy expenditure in metabolic equivalents (kcal/kg/week) was estimated using the Modifiable Activity Questionnaire [24]. The questionnaire was modified by adding hula dancing, surfing, fishing from a boat, fishing from the shore, and skin diving. Energy expenditures were estimated using the American College of Sports Medicine’s Compendium of Physical Activities [25]. Both the past week’s and past month’s average weekly energy expenditure were estimated and transformed taking the natural logarithm to normalize the data. To reduce the effects of recall bias, we only used past week’s energy expenditure in this analysis. Participants were then classified as active if they expended at least 45.75 MET/hrs per week, the cut-point for those in the highest quartile of activity.

Resting heart rates were recorded by electrocardiogram after the participants were resting in a quiet room for at least 10 minutes. Resting heart rate was also dichotomized using the population median (63 bpm) as the cut-point. Finally, each participant was further categorized as less active/high RHR, active/high RHR, less active/low RHR, and active/low RHR.

### Statistical analyses

Bivariate analyses were first performed between each of the outcomes of interest (HOMA-IR and ISR), primary risk factors (RHR and PA), and potential confounding variables. Linear regression was used to estimate associations between continuous variables, and analysis of variance (ANOVA) was used to test for differences between groups for categorical variables, such as gender and ethnic group. After potential confounders were identified, associations between each of the outcomes and primary risk factors were estimated by multiple linear regression using the General Linear Model method. All analyses were performed using JMP statistical software (SAS Institute, Gary, NC).

## Results

The population characteristics of KHS by ethnic ancestry are shown in Table 1. Ethnic groups differed significantly by BMI, WHR, and age. Both outcomes, HOMA-IR and ISR, differed by ethnic group, as did both primary risk factors, PA and RHR. Thus, ethnicity was identified as a potential confounding variable.

**Table 1:** **Population characteristics, Kohala Health Study, 1997-2001(means and standard deviations)**

**Ethnic group**	**Caucasian**	**Filipino**	**Hawaiian/Part-Hawaiian**	**Japanese**	**Other/mixed**	**P-value**
N	291	167	469	169	231	
Age	49.4 (12.5)	53.2 (16.7)	43.4 (14.4)	58.0 (16.6)	46.3 (16.5)	<0.001
Gender (% female)	49.5	59.9	55.2	52.1	55.4	Not Significant
Body mass index	25.5 (5.2)	25.8 (5.1)	30.9 (8.8)	25.6 (4.2)	27.1 (5.4	<0.001
HOMA-IR	3.83 (2.72)	4.25 (2.40)	5.15(3.35)	4.31 (2.28)	4.16(2.79)	<0.001
Insulin secretion ratio	0.80 (1.49)	1.06 (1.25)	1.50 (4.04)	0.80 (0.81)	1.07 (2.81)	<0.01
Resting heart rate	61.4 (10.4)	66.2 (10.3)	64.3 (9.0)	65.3 (9.2)	64.8 (9.7)	<0.001
METS/week	41.4 (50.1)	25.0 (26.8)	38.2 (52.3)	30.7 (37.7)	38.3 (50.3)	<0.01

Other correlates of the primary outcomes, HOMA-IR and ISR, are shown in Table 2. Neither outcome differed significantly by gender. While HOMA-IR was associated only with BMI and WHR, ISR was associated with age and BMI, but not WHR.

**Table 2:** **Linear regression estimates of correlates of insulin resistance estimated by natural logarithm of HOMA-IR and insulin secretion ratio, Kohala health study, 1997-2001**

	**HOMA-IR**				**Insulin secretion ratio**			
**Variable**	**Estimate**	**Standard error**	**F ratio**	**Prob > F**	**Estimate**	**Standard error**	**F ratio**	**Prob > F**
Age	0.008	0.005	2.28	0.13	−0.015	0.082	10.3	<0.0001
Body mass index	0.178	0.011	271.97	<0.0001	0.042	0.012	10.6	0.0014
Waist/hip	6.53	1.086	36.13	<0.0001	−0.384	1.090	0.12	0.72
Natural log of past week leisure time physical activity (MET hrs/wk)	−0.066	0.068	15.28	<0.0001	−0.022	0.026	0.75	0.40
Resting heart rate	0.012	0.002	45.52	<0.0001	0.001	0.003	0.16	0.69
Gender (Means)								
Male	4.49	0.119	0.79	0.79	1.024	0.116	1.25	0.26
Female	4.45	0.110			1.200	0.107		

Table 3 shows the correlates of the primary risk factors, PA and HR. Age was positively associated with HR and inversely associated with PA. PA was higher and HR was lower among males. Although increasing BMI was associated with increasing HR, it was not associated with PA. In contrast, WHR was inversely associated with PA, but not significantly associated with HR. Finally, as shown in Table 3, HR and PA had a significant inverse association.

**Table 3:** **Linear regression estimates of correlates of resting heart rate and past week’s leisure time physical activity, Kohala health study, 1997-2001**

	**Resting heart rate**				**Leisure time physical activity**			
**Variable**	**Estimate**	**Standard error**	**F ratio**	**Prob > F**	**Estimate**	**Standard error**	**F ratio**	**Prob > F**
Age	0.055	0.017	10.52	<0.0001	−0.289	0.085	11.64	0.0007
Body mass index	0.227	0.043	47.39	<0.0001	−0.113	0.220	0.2661	0.6060
Waist/hip	6.205	3.75	2.74	0.09	50.457	18.537	2.72	0.0066
Resting heart rate	-	-	-	-	−0.561	0.138	16.65	<0.0001
Gender (Means)								
Male	63.02	0.394	14.44	0.0002	45.312	1.926	41.49	<.0001
Female	65.06	0.367			28.289	1.809		

Next, multi-variable linear regression was performed using the General Linear Model methods. Covariates for the outcome HOMA-IR included ethnic group, BMI, WHR, and the primary risk factors, RHR and PA (Table 4). With the inclusion of the covariates, ethnic group was no longer significantly associated with HOMA-IR. Both HR and PA were independently and significantly associated with HOMA-IR. In the regression model for ISR (Table 5), with the addition of the primary outcomes, covariates included ethnic group, age, and BMI. In contrast to HOMA-IR, neither of the primary outcomes were significantly associated with ISR, and none of the covariates appeared to explain the ethnic group differences in ISR.

**Table 4:** **Regression estimates of the effect of resting heart rate and past week’s leisure time activity on insulin resistance estimated by the natural logarithm of HOMA-IR, using the General linear model approach**

**Source**	**Degrees of freedom**	**Sum of squares**	**F ratio**	**Prob > F**
Ethnic group	4	1.48	1.23	0.2969
Body mass index	1	48.53	160.71	<.0001
Waist/hip	1	2.28	7.56	0.0061
Natural log of past week leisure time physical activity (MET hrs/wk)	1	4.98	16.49	<.0001
Resting heart rate	1	5.12	16.96	<.0001

**Table 5:** **Regression estimates of the effect of resting heart rate and past week’s leisure time activity on beta-cell function estimated by the insulin secretion ratio, using the General linear model approach**

**Variable**	**Degrees of freedom**	**Sum of squares**	**F ratio**	**Prob > F**
Ethnic group	4	8.26	2.73	0.0282
Age	1	49.31	65.14	<.0001
Body mass index	1	21.92	28.96	<.0001
Natural log of past week leisure time physical activity (MET hrs/wk)	1	2.19	2.90	0.0891
Resting heart rate	1	0.019	0.025	0.8752

In the final analysis (Table 6), when PA and HR were dichotomized and combined as a categorical variable, post hoc analysis revealed that for those with high RHR, there was no significant difference in insulin resistance. However, both groups of participants with low RHR had significantly lower levels of insulin resistance compared to participants with high RHR. Moreover, those in the high activity group with low RHR were observed to have significantly lower levels of IR than sedentary persons with lower PA.

**Table 6:** **Comparison of least squared means of the natural logarithm of HOMA-IR by heart rate and physical activity levels combined, adjusted for age, ethnic group and BMI**

**Level**	**Least squares mean**	**Standard error**	Tukey’s HSD*
Less active and low resting heart rate	1.39	0.026	A
Active and high resting heart rate	1.32	0.047	A B
Less active and low resting heart rate	1.28	0.027	B
Active and low resting heart rate	1.11	0.042	C

*Levels not sharing the same letter are significantly different.

## Discussion

In summary, after adjusting for possible confounding variables, both PA and RHR had significant, independent associations with HOMA-IR and, in combination with other risk factors, it explained the initial ethnic differences in insulin resistance. This was not the case with first phase insulin secretion estimated by ISR, with which neither risk factor had a significant association. Further analysis revealed that the greatest effect on insulin resistance was seen in participants with both high levels of activity and low RHR, followed by less active participants with similarly low RHR. Although a slight reduction in insulin resistance was observed among those who had high RHR but were active, this difference was not statistically significant.

The question arises of the mechanism driving the association between RHR and IR. RHR is commonly used in epidemiological studies as a marker of cardiovascular fitness, and is commonly observed to be low in athletes and participants in endurance exercise, probably due to increased stroke volume [26]. The independent role of cardiovascular fitness on CVD risk has been noted by other researchers [27,28]. Our findings suggest this may be in part due to the effect of cardiovascular fitness on insulin sensitivity. Our observation that this effect is independent of high levels of PA may be due to two possible factors. First, low RHR may be indicative of cardiovascular fitness determined by genetic factors [29-32], and fitness then in turn could conceivably be associated with improved insulin sensitivity. Therefore, the independent association of fitness and insulin sensitivity may be a result of common genetic determinants. Second, the effect of low RHR may reflect differences in cardiovascular fitness determined by intensity of PA that was not captured fully by the use of self-report measures or it may also reflect a limitation of the use of MET hours per week, as a measure of energy expenditure, which is a function of both duration as well as intensity. Lin et al. [33] reported that when examined together, cardiovascular fitness measured by VO(2)max, but not self-reported PA, had a significant association with insulin resistance. In contrast, Ekelund et al.[34] found that PA energy expenditure, measured objectively by monitored heart rates, was predictive of progression of metabolic syndrome, while VO(2)max was not independently predictive.

Another explanation of the independent role of RHR on insulin sensitivity may be that the sympathetic nervous system (SNS) may serve as a common pathway for both factors. Both obesity and hypertension have been associated with a relative increased sympathetic tone that might reflect a disturbance in the parasympathetic regulation of the heart [35,36]. Some investigators have suggested increased insulin resistance and the accompanying hyperinsulinemia results in stimulation of the SNS thus increasing resting heart rate. The IRAS study demonstrated that HR was prospectively predictive of both insulin resistance and decreased insulin secretion [20]. However, others have suggested autonomic dysfunction may precede insulin resistance [37,38]. In all likelihood, the relationship between the autonomic nervous system and insulin resistance is complex and bidirectional. Also, it should be noted that the SNS is also involved in insulin release; however, we observed no association between RHR and ISR.

There are several limitations to the present study. First, as for all cross sectional data, no causation can be attributed. This limitation is especially important as it limits our interpretation of the role of RHR on insulin sensitivity. If it is an indicator of autonomic function, then it is possible that insulin resistance is just as likely to have affected RHR as the converse scenario. If RHR is an indicator of fitness, we cannot be certain that the association is not due to common genetic factors affecting both fitness and insulin sensitivity.

Another limitation alluded to above, is the use of self-report to estimate actual PA. Error in recall can be expected to lead to exposure misclassification. Although misclassification of PA is most likely non-differential with regards to the outcome, the result would be attenuation of the observed association. Since physical activity has been reported to be more strongly influenced by exercise intensity [39], RHR may then be acting as a proxy variable that functions to reduce the error and misclassification or better characterizes the intensity of physical activity.

Finally, another limitation of this study was that medication use was not available for this analysis. While the observation that lower activity/low RHR was associated with better insulin sensitivity than higher activity/high RHR, it may be due to independent effects of fitness as explained above. It also may be in part due to the effects of medication use. While beta- specific blockers have been shown to be associated with poorer insulin sensitivity, non-specific beta-blockers that also act on alpha receptors, and calcium channel blockers have been shown to increase insulin sensitivity [40]. Hence, the lowered RHR among the less active participants could be in part due to the effects of medications. To better ascertain if low RHR was a result of medication use, we performed a post hoc medical history review of each participant who had both a low level of physical activity and low RHR. Since only two participants in this category reported using either a beta-blocker or calcium channel blocker, it is unlikely that medication use could explain the observed effect on insulin resistance.

## Conclusion

Regardless of the limitations of the primary risk factors, the findings of this study support the notion proposed by Williams [27,28] that fitness might be an independent predictor of the development of CVD risk factors and CVD mortality and should be considered as a screening factor for individuals at risk. Our findings also have an important methodological implication, that use of RHR could serve as a useful proxy for either fitness or of physical activity that may not be completely captured by use of self-reported physical activity. Future research should focus on elucidating the role of fitness on insulin resistance, to what degree fitness can be modified, and whether or not interventions targeted at improving fitness level can be effective and feasible.
